# Serum miR-26a as a diagnostic and prognostic biomarker in cholangiocarcinoma

**DOI:** 10.18632/oncotarget.4072

**Published:** 2015-05-29

**Authors:** Li-Juan Wang, Kai-Liang Zhang, Ning Zhang, Xiang-Wei Ma, Su-Wen Yan, Dong-Hua Cao, Sheng-Jia Shi

**Affiliations:** ^1^ Department of Oncology, The First Affiliated Hospital of Medical College of Xi'an Jiaotong University, Xi'an, 710061, Shaanxi Province, P.R. China; ^2^ Deparment of Orthopedics, No. 88 Hospital of PLA, Tai'an, 271000, Shandong Province, P.R. China; ^3^ Deparment of Aristogenesis, No. 202 Hospital of PLA, Shenyang, 110003, Liaoning Province, P.R. China

**Keywords:** serum miR-26a, diagnostic, prognosis, cholangiocarcinoma

## Abstract

In order to determine the diagnostic and prognostic value of miR-26a in Cholangiocarcinoma (CCA), we compared miR-26a levels in serum from 66 CCA patients and 66 healthy controls, which was followed by serum analysis between the pre-operative serum and post-operative serum of these CCA patients. We found the concentration levels of miR-26a in serum of CCA patients were significantly higher than that from healthy controls (*P* < 0.01). Furthermore, the concentration levels of miR-26a in the post-operative serum were significantly reduced when compared to the pre-operative serum (*P* < 0.001). High miR-26a in serum was correlated significantly with clinical stage, distant metastasis, differentiation status, and poor survival of CCA patients. More importantly, serum miR-26a was an independent prognostic marker for CCA. In conclusion, our results suggested that miR-26a in serum might be a potential and useful noninvasive biomarker for the early detection of CCA.

## INTRODUCTION

Cholangiocarcinoma (CCA), the second most common primary hepatic malignancy [1], is epithelial cancer of biliary tree [2]. Though the rate of extrahepatic CCA (EHCC), has remained stable, the incidence of intrahepatic CCA (IHCC) has doubled in the last 40 years [3]. CCAs are usually diagnosed in the late stages, and the median survival for those with advanced or metastatic CCA is usually measured in months, and the overall five-year survival is less than 5% [4]. Surgical resection, determined by the location, extent of disease and involvement of surrounding tissues, offers the only chance of potentially curative treatment and long-term cure [5, 6]. Unfortunately, the respectability rates are generally low, survival resection varies widely between centers (range 23–50% at 5 years) [7], and the majority of patients with resected CCA still develop recurrent or metastatic disease. The current serum tumor marker carbohydrate antibody 19-9 (CA19-9) has the limitation of low sensitivity and specificity. A paucity of disease-specific symptoms and the low specificity of currently diagnostic techniques in the early stages [2] made the development of minimally or noninvasive, highly sensitive and specific CCA biomarkers become an urgent demand.

MiRNAs are noncoding RNAs 18–25 nucleotides in length, which are recently demonstrated to play a major role in the regulation of virtually all cellular processes [8] and gene expression [9, 10] by binding to the target sites of miRNAs. Recently, several studies have shown that aberrant miRNAs expression is associated with the genesis and homeostasis of CCA [11, 12]. Subsequent studies demonstrated that the expression of miR-26a, miR-21, miR-29, miR-124, miR-370, miR-373, miR-182, miR-27b, miR-let7b, miR-221, miR-181a and miR-494 were linked to cholangiocarcinogenesis [13–20]. But the major hinder in using miRNAs as a diagnostic biomarker in CCA is that all the mentioned miRNAs are detected in CCA cell lines or human tissues, which are invasive and not conductive to popularity.

Recently, several studies have shown that miRNAs in human serum could be detected as biomarkers to diagnose several cancers with high sensitivity and specificity [21–27]. As expected, numerous publication have reported that serum miRNAs had different levels between tumor patients and healthy controls [23, 28–31] and might serve as stable blood-based biomarkers [32–34] in various cancer diseases, which further highlighted the potential of circulating miRNAs as non-invasive diagnostic biomarkers and prognostication against cancerous diseases. Although the origins and physiologic functions of cell-free miRNAs in the serum remain to be fully elucidated, expression profiling of miRNAs in different cancers should be identified to exploit these molecules as potential diagnostic and prognostic biomarkers. MiR-26a has been proven to have an aberrantly expression in the tissue of CCA patients and associated with genesis and homeostasis of CCA [16]. However there is still no study to investigate the expression profiling, diagnostic and prognostic significance of serum miR26a in CCA patients.

In the present study, we systematically investigated the role of miR-26a in CCA by a two-phase study. In the first phase, we performed quantitative analyses of miR-26a in a subset of serum samples from CCA patients and healthy control subjects to determine the feasibility of their detection in the circulation. In the second phase, we evaluated the clinical significance of miR-26a as potential biomarkers for diagnosis and prognosis of CCA patients.

## RESULTS

### Relative expression of serum miR-26a in CCA patients

**Figure 1:**
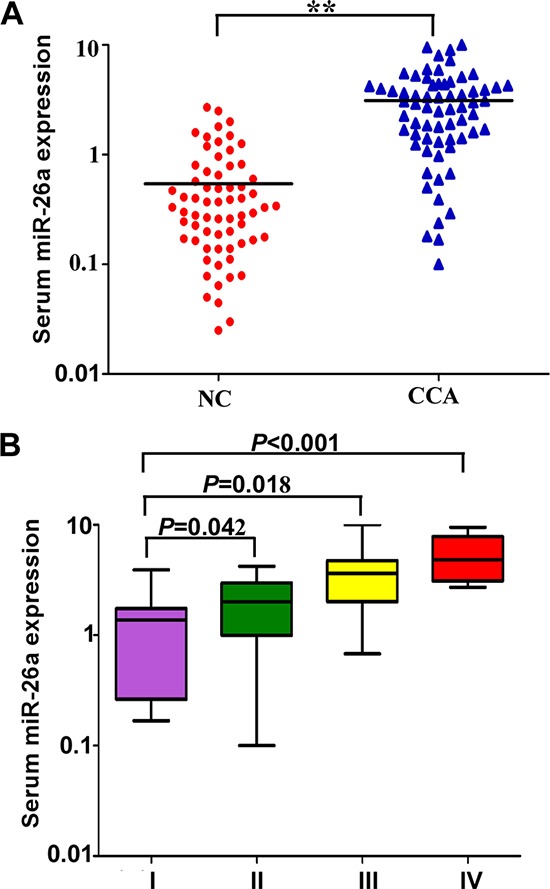
Expression of miR-26a in the serum of CCA patients **A.** The levels of serum miR-26a in normal controls and CCA patients. **B.** The level of serum miR-26a in different TNM stages of CCA patients. Two-tailed Student's *t* test was used to analyze the significant differences. *P < 0.05.

We investigated the level of serum miR-26a in CCA patients. We found the relative expression of serum miR-26a in 66 CCA patients was significantly higher than that from healthy controls (*P* < 0.01) (Figure 1A). Furthermore, to identify whether the expression of serum miR-26a was associated with clinical stage of CCA patients, the expression levels of serum miR-26a from the same 66 CCA patients were analyzed according to their clinical stage. Our data indicated that the expression levels of serum miR-26a were significantly increased as the TNM stage increased, and the expression of serum miR-26a was statistically significantly lower in TNM I stage patients than stage II, III or IV patients (Both *P* < 0.05) (Figure 1B).

### Serum miR-26a is a potential diagnostic biomarker for CCA patients

**Figure 2:**
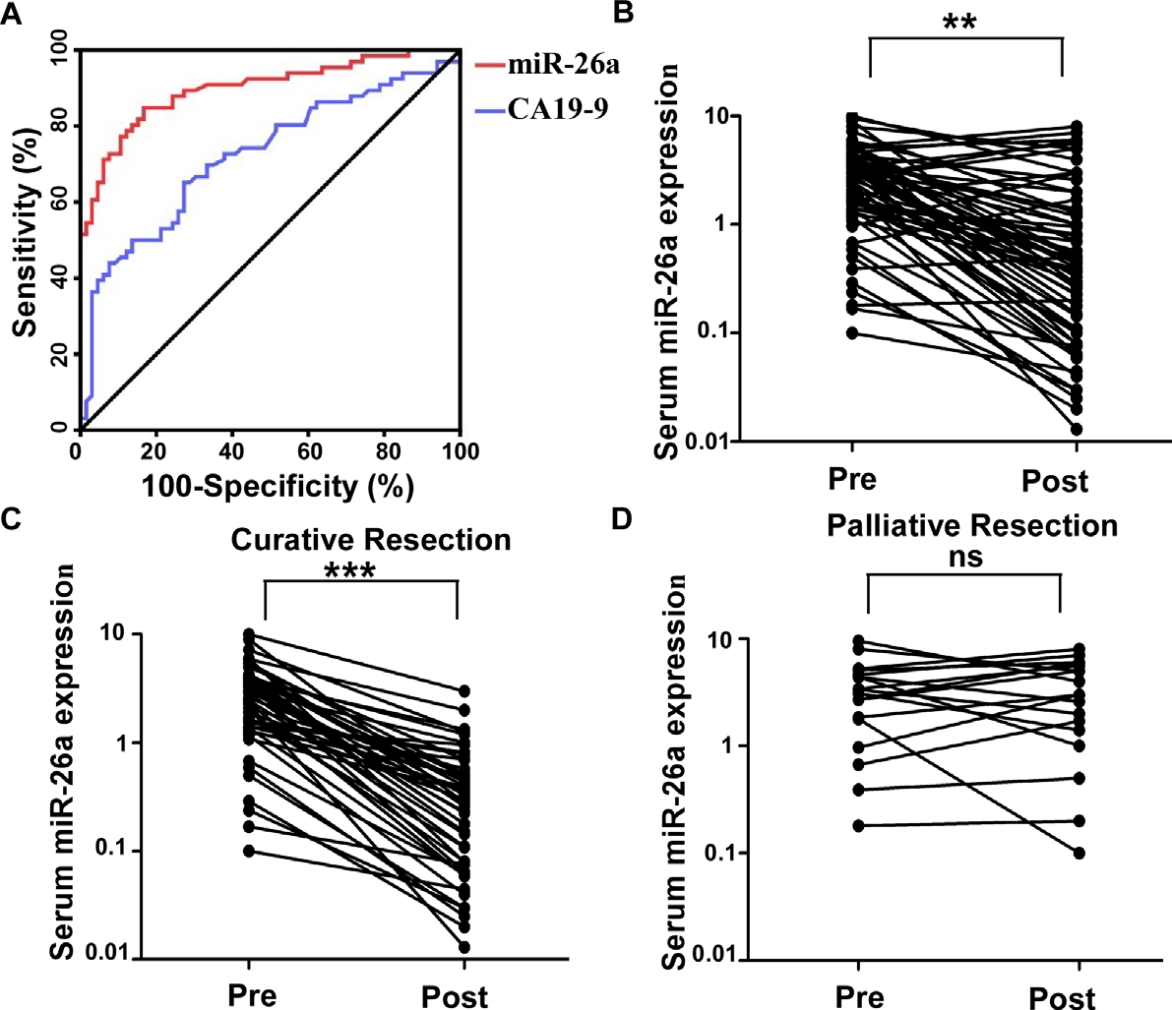
Serum miR-26a is a potential diagnostic biomarker for CCA patients **A.** Serum miR-26a yielded an area under the curve (AUC) value of 0.899, with 84.8% sensitivity and 81.8% specificity in distinguishing CCA from normal control subjects. **B.** Comparison of serum miR-26a levels from all CCA patients (*n* = 66). **C.** Comparison of serum miR-26a levels in 48 CCA patients who underwent potentially curative surgeries. **D.** Comparison of serum miR-26a in 18 CCA patients who underwent palliative resections. Two-tailed Student's *t* test was used to analyze the significant differences. *P < 0.05.

Based on these previous results, we focused our study on the efficacy of serum miR-26a as a diagnostic biomarker in patients with CCA in the following experiments. So we generated ROC curves to assess the potential usefulness of serum miR-26a as a noninvasive biomarker for early diagnosis of CCA. Our ROC analyses revealed that serum miR-26a levels were robust in discriminating patients with CCA form healthy control subjects with an AUC value of 0.899, which was significantly higher than that of conventional CAA marker CA19–9 (AUC value = 0.723) (Figure 2A). Using a cutoff value of 0.96, the sensitivity, specificity, and positive and negative predictive values were 84.8%, 81.8%, 82.3% and 84.4%, respectively, to identify a patient with CCA.

### Alterations in serum miR-26a expression levels in patients with CCA

Thereafter, we analyzed paired pre- and postoperative serum samples in the subset of 66 CCA patients who underwent surgical resection of their tumor. In the 66 CCA patients, 48 underwent potentially curative resection, whereas 18 had multiple hepatic metastases and underwent palliative resection. We found that serum levels of miR-26a statistically significantly plummeted after surgery in the same subset of patients (*P* < 0.01; Figure 2B). However, when data were analyzed based on potentially curative or palliative surgeries, postoperative reductions in serum miR-26a levels occurred exclusively among patients with potentially curative surgeries (*P* < 0.001; Figure 2C). Contrariwise, no statistically significant difference were observed in miR-26a levels before or after surgery in patients with palliative resections (Figure 2D). Taken together, these data underscore the importance of serum miR-26a expression as a highly specific biomarker for the diagnosis of CAA.

### Correlation of serum miR-26a of CCA patients with clinicopathological factors

Next, we asked whether serum miR-26a expression was correlated with clinicopathological characteristics of patients with CCA. As shown in Table 1, miR-26a was significantly up-regulated in CCA patients with advanced clinical stage (*P* = 0.005), lymph vessel infiltration (*P* = 0.007), metastasis status (*P* = 0.036), and differentiation status (*P* = 0.013). However, there was no correlation of miR-26a expression with other clinical features, such as age, gender, and invasion depth (all at *P* > 0.05, respectively).

**Table 1:** Association between serum miR-26a expression and clinicopathological variables of CCA patients

Clinicopathological features	No.	Relative miR-26a expression (mean ± SD)	*P*
**Age at diagnosis (years)**			
**≤ 60**	32	3.09 ± 2.27	0.654
**> 60**	34	3.13 ± 2.32	
**Gender**			
**Male**	38	3.04 ± 2.38	0.609
**Female**	28	3.20 ± 2.18	
**Clinical stage at diagnosis**			
**I-II**	29	2.29 ± 1.44	0.005^*^
**III-IV**	37	3.75 ± 2.61	
**Invasion depth**			
**T1-T2**	27	3.18 ± 2.36	0.776
**T3-T4**	39	3.67 ± 2.60	
**Lymph vessel infiltration**			
**Yes**	24	4.14 ± 2.17	0.007^*^
**No**	42	2.71 ± 2.04	
**Distant metastasis**			
**Yes**	10	4.50 ± 2.87	0.036^*^
**No**	56	2.86 ± 2.10	
**Differentiation**			
**Well**	39	2.54 ± 1.79	0.013^*^
**Moderate/Poor**	27	3.94 ± 2.67	

* Significant relation of clinical factors with overall survival

### Association of serum miR-26a expression with survival and prognosis in patients with CCA

**Figure 3:**
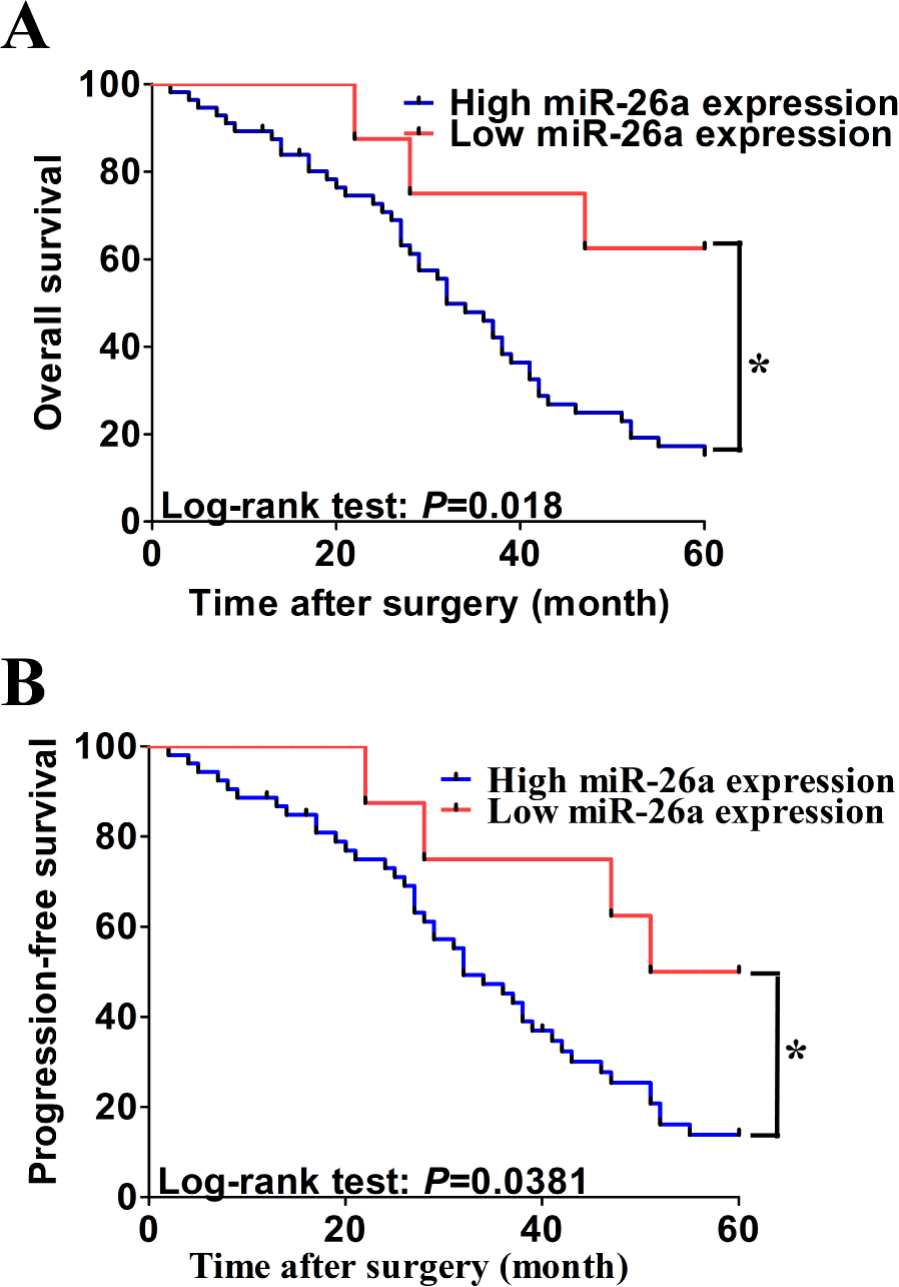
Association of serum miR-26a expression with progression-free and overall survival **A.** and **B.** Kaplan-Meier graphs representing the probabilities of progression-free and overall survival in CCA patients according to expression level of miR-26a. Two-tailed Student's *t* test was used to analyze the significant differences. *P < 0.05.

To determine the prognostic value of serum miR-26a expression in CCA, we analyzed the relationship between the serum miR-26a and clinical outcome. The relationships between miR-26a expression and overall survival or progression-free survival were investigated using Kaplan-Meier analysis and log-rank test. Statistically significant difference\ in overall survival and progression-free survival was found between the high serum miR-26a expression group and low serum miR-26a group (Figure 3A and 3B; log-rank test: *P* = 0.018 and *P* = 0.0381, respectively). The patients with high serum miR-26a expression tended to have shorter overall and progression-free survival time when compared to patients with low serum miR-26a expression. Univariate analysis identified clinical stage, distant metastasis, lymph vessel infiltration, differentiation status and high expression of serum miR-26a as poor prognosticators for overall survival and progression-free survival (all *P* < 0.05), whereas age, gender and invasion depth were not significantly associated with overall survival and progression-free survival (Tables 2 and 3). To test whether the prognostic value of high serum miR-26a expression was independent of other risk factors for poor overall and progression-free survival, a multivariate analysis was performed using a Cox proportional hazard model. Multivariate analyses including age, gender, tumor location, tumor stage, serum miR-26a expression, differentiation status, lymph vessel infiltration, invasion depth and distant metastasis demonstrated that high serum miR-26a expression was an independent predictor for poor overall and progression-free survival in CCA patients (HR = 3.461, CI = 1.331–5.364, *P* = 0.013 and HR = 4.226, CI = 1.415–10.321, *P* < 0.001 respectively). Statistically significant results were also obtained for advanced clinical stage, distant metastasis, and differentiation status, whereas all other parameters were not significant independent prognostic maker for overall and progression-free survival (Tables 2 and Table 3).

**Table 2:** Univariate and Multivariate analysis of clinical parameters in relation to overall survival

	*Univariate*		*Multivariate*	
Variables	*HR (95%CI)*	*P*	*R (95%CI)*	*P*
**miR-26a expression**	3.732 (1.814–6.421)	0.031^*^	3.461(1.331–5.364)	0.013^*^
**Age at diagnosis**	1.515 (0.574–1.842)	0.583	1.215(0.609–2.074)	0.437
**Gender**	1.936 (0.762–2.452)	0.678	1.548 (0.652–2.115)	0.547
**Clinical stage at diagnosis**	3.547 (1.427–4.416)	0.015^*^	3.112 (1.268–3.709)	<0.001^*^
**Invasion depth**	2.615 (0.894–3.431)	0.106	2.314 (0.852–2.472)	0.0891
**Lymph vessel infiltration**	3.462 (2.134–5.383)	0.031^*^	2.953 (2.141–3.458)	0.007^*^
**Distant metastasis**	2.784 (1.487–4.125)	0.017^*^	3.456 (1.424–6.135)	0.008^*^
**Differentiation**	4.193 (2.216–7.146)	0.002^*^	2.986 (1.897–4.116)	0.017^*^

CI: confidence interval; HR: hazard ratio

* Significant relation of clinical factors with overall survival

**Table 3:** Univariate and Multivariate analysis of clinical parameters in relation to progression-free survival

	*Univariate*		*Multivariate*	
Variables	*HR (95%CI)*	*P*	*HR (95%CI)*	*P*
**miR-26a expression**	3.115 (1.714–4.211)	0.008^*^	4.226 (1.415–10.321)	< 0.001^*^
**Age at diagnosis**	1.724 (0.566–2.341)	0.731	1. 578 (0.628–1.874)	0.416
**Gender**	2.154 (0.763–3.215)	0.329	1.984 (0.458–2.409)	0.471
**Clinical stage at diagnosis**	4.252 (1.472–6.179)	0.004^*^	3.241 (1.762–5.745)	< 0.001^*^
**Invasion depth**	2.112 (0.751–3.14)	0.195	2.154 (0.793–2.853)	0.217
**Lymph vessel infiltration**	2.791 (1.415–4.117)	0.042^*^	2.117 (1.317–4.561)	0.017^*^
**Distant metastasis**	5.104 (1.998–10.179)	< 0.001^*^	4.219 (2.326–7.619)	< 0.001^*^
**Differentiation**	3.146 (1.342–5.145)	0.042^*^	2.754 (1.532–4.156)	0.014^*^

CI: confidence interval; HR: hazard ratio;

* Significant relation of clinical parameters with progression-free survival

## DISCUSSION

Aberrant miRNA expression patterns have been described in the tissues or cells of various cancers, and the alterations in miRNAs expression are highly correlated with progression and prognosis of human malignant diseases [35–37]. However the major hinder in using miRNAs as a non-invasively diagnostic biomarker and prognostic factor is that we used to speculate miRNAs could not keep intact in serum, as tremendous RNases are existed in circulation [38, 39]. However, this opinion has been changed since Mitchell et al. firstly detected tumor-derived miRNA in circulation in year 2008 [34]. Their presence in the blood has attracted the attention of researchers due to their potential use as non-invasive blood biomarkers [32]. Nowadays, circulating miRNAs in serum have been detected in many solid cancers as well as digestive tract cancers and have been proven to be a valuable diagnostic biomarker with minimally invasive, including prostate cancer, breast cancer, pancreatic cancer, gastric cancer, etc [28, 31, 34, 40–42]. The mainly poor prognosis associated with CCA is lack of minimally invasive, early detection tests, therefore identifying a circulating miRNA in serum as biomarker for CCA seems to be very urgent and important.

In the current study, we identified the diagnostic and prognostic role of serum miR-26a in CCA patients. We identified the diagnostic role of serum miR-26a in CCA by two steps. At the beginning, we found the relative expression of serum miR-26a in CCA patients was significantly higher than that from healthy controls, and the expression of serum miR-26a correlated significantly with TNM stage of CCA patients. In the last, we demonstrated the potential role of serum miR-26a in the early detection of CCA by yield AUC curves, and compare the AUC curves of serum miR-26a and conventional CAA marker CA19–9. We found the AUC value of serum miR-26a was even higher than CA19–9, which indicated the potential diagnostic role of serum miR-26a in early detection of CAA. Taken together, we validated that the extraction of RNA and identification of miR-26a in the serum of individuals diagnosed with CCA is feasible and offered the first description that miR-26a could be an effective diagnostic biomarker with high sensitivity and specificity for CCA.

Another important finding of our study is that serum miR-26a expression also serves as a prognostic biomarker for CCA. Our results are consistent with previous studies which demonstrated miR-26a could promote CCA cell growth and might be targets for prevention or treatment of CCA [16]. We identified the prognostic role of serum miR-26a in CAA by three steps. In the beginning, we found the expression of serum miR-26a was significantly correlated with adverse clinicopathological factors. Then, we found serum miR-26a was also significantly correlated with progression-free and overall survival of CCA patients by Kaplan-Meier and Univariate analysis. In the last, we identified serum miR-26a was an independent prognostic biomarker for patients with CAA by multivariate analysis. Our findings that high levels of serum miR-26a indicate a poor prognosis in patients with CAA are also an important step forward in the further identification of a noninvasive biomarker for CCA. Taken together, our study demonstrates that serum miR-26a might not only diagnostic biomarker for CCA but also help predict metastases or tumor recurrence with higher accuracy.

The role of miR-26a is extremely different in different solid cancer. Down-regulated miR-26a was reported play important role in the progression of tumor and severed as a potential tumor suppressor in several distinct cancer types [43, 44]. But it was proved to act as oncogenic miRNA in ovarian cancer, hepatocellular carcinoma, chronic lymphocytic leukemia and CCA [16, 45–47]. Especially, miR-26a had been proven to be a secretory miRNA and could distinguished patients from healthy controls in ovary cancer [45], which indicated serum miR-26a could be powerful biomarkers in cancer diagnostics. In cohort with the previous study, our study also proved miR-26a was an oncogenic miRNA in CCA, and the level of serum miR-26a was a powerful diagnostic and prognostic biomarker for CAA patients. But considering the different roles of miR-26a in different cancers, more study are urgent needed to validate the possibility of serum miR-26a applied as a biomarker in clinical setting.

Although our current study indicate serum miR-26a maybe a promising screening tool for CCA, we acknowledge two potential limitations of using miR-26a as a single biomarker for the early detection of CCA. First, circulating expression of miR-26a has been described in many solid cancers besides CCA, such as hepatocellular carcinoma [48], ovarian cancer [49], and renal cell carcinoma [50]. As a consequence, it might be challenging to differentiate whether circulating miR-26a expression is specifically associated with CCA itself or if this is a common phenomenon that manifests during progression of any cancer as a result of perturbations in the host immune response [51]. Second, although it is highly unlikely to have a substantial impact, use of serum miR-26a expression levels as a diagnostic and prognostic biomarker must be validated in diverse ethnic populations because the clinical specimens analyzed in our study were solely from patients of Chinese origin.

In conclusion, we proved serum miR-26a was an effective diagnostic biomarker in CCA patients. Besides, we also demonstrated serum miR-26a levels were more frequently elevated in CCA patients with adverse clinical stage and presence of distant metastasis. Multivariate survival analyses demonstrated that serum miR-26a was an independent prognostic factor for both progression-free and overall survival in CCA. Our results provide compelling evidence for the potential usefulness of serum miR-26a as a noninvasive screening tool and effective prognostic tool in patients with CCA.

## MATERIALS AND METHODS

### Patients and specimens

Serum-based specimen collection and studies were approved by the Research Ethics Committee of No.202 hospital of PLA. All patients provided written consent and indicated willingness to donate their blood samples for research. A total of 66 patients were enrolled in this study. 48 patients received curative resection, and 18 patients received palliative resection at No.202 hospital of PLA. (Shenyang, China) from 2007 to 2009. All patients enrolled didn't receive radiotherapy or chemotherapy before operation. All tumors were clinically and histologically diagnosed as cholangiocarcinoma. Inclusion criteria for all cases included: (i) unambiguous histology and absence of mixed tumor types; (ii) absence of any treatment prior to surgery; The clinicopahtological characteristics of patients are given in Table 1.

### Cell culture

QBC939 and RBE human CCA cell lines were cultured in 1640 medium with 10% foetal bovine serum (FBS) (Hyclone, Logan, UT, USA), and were incubated at 37°C in a humidified atmosphere containing 5% carbon dioxide. To determine the secretory potential of miR-26a, a fraction of the culture medium of QBC939 and RBE cell lines was collected at 0, 12, 24, 36 and 48 hours after the initial seeding of cells in 10-cm dishes.

### RNA extraction and quantitative real-time polymerase chain reaction (qRT-PCR)

For miRNA quantification, total miRNA was extracted from the cells using miRNeasy RNA isolation Kit (Qiagen, Valencia, CA, USA), according to the manufacturer's instructions. Synthetic cel-miR-39 (QIAGEN, 219610) was added as a spike-in control miRNA into each sample to normalize the sample-to-sample variation in the RNA isolation step and detect purification efficiency [31]. TaqMan miRNA qRT-PCR (Applied Biosystems, Foster City, CA, USA) were used to detect and quantify miRNA expression. Data were analyzed with 7500 software v.2.0.1 (Applied Biosystems, Foster City, CA, USA), with the automatic Ct setting for adapting baseline and threshold for Ct determination. Each sample was examined in triplicate and the amounts of PCR products produced were nonneoplasticized to RNU6B.

### Statistical analyses

Statistical analysis was performed using IBM SPSS statistical software (version 20.0) (International Business Machines Corporation, Armonk, NY, USA). Mann-Whitney U analyses of variance were used to evaluate statistical differences in serum miRNA expression between unpaired groups. The Wilcoxon test was used to compare miR-26a expression in paired serum samples obtained before surgical tumor resection and 7 days after surgical tumor resection. Receiver operating characteristic (ROC) analysis was performed to determine the diagnostic performance of miR-26a expression levels in distinguishing patients with CCA form healthy control subjects. Sensitivity against 100% minus specificity was plotted each cutoff threshold, and the area under the curve (AUC) values that reflect the probability of correctly identifying CCA patients from control subjects were computed. The optimal cutoff thresholds for diagnosis were obtained by Youden index. By using the optimal cutoff value, sensitivity, specificity, and positive and negative predictive values were calculated. Survival curves were estimated using the Kaplan-Meier method, and distributions were evaluated by the long-rank test. Cox proportional hazard models of factors related to survival were used to calculate HRs and identify the factors that affect survival. The differences in characteristics between the 2 groups were examined by the (2 test and Fisher's exact test. All *P*-values were determined from 2-sided tests, and statistical significance was based on a *P*-value of 0.05.

## Abbreviations

CCA: Cholangiocarcinoma
miRNAs: microRNAs
qRT-PCR: Quantitative Real-time Polymerase Chain Reaction
ROC: Receiver operating characteristic
AUC: Area under the curve
IHCC: Intrahepatic CCA
EHCC: Extrahepatic CCA
